# Supplementary material for: Obstructive Sleep Apnea and Circulating Biomarkers of Oxidative Stress: A Cross-Sectional Study
Source: Antioxidants (Basel). 2020 Jun 2;9(6):476. doi: 10.3390/antiox9060476 (PMC7346202; doi:10.3390/antiox9060476)
Supplement: Supplementary file 1 [file antioxidants-09-00476-s001.pdf]

Supplemental Information

This supplemental material provides additional information about the assays used in the main manuscript.

8-isoprostane

All samples were done in duplicate and fitted to their own plate's standard curve. Each plate had 40 samples. The total of samples outside the detected range was 2% (11 out of 402). Of those, 8 presented with a value higher than the upper limit of the standard curve, and 3 lower.

Table S1. Technical Data on 8-isoprostane Assays.

Plate	Curve Fit R^2	CV of Standard Curve (%)	CV of Samples (%)	Samples Above Detectable Range (count)	Samples Below Detectable Range (count)
Pilot	1.000	4.928	18.28	0	0
1	0.999	3.816	15.81	0	0
2	0.999	4.071	33.85	0	0
3	0.999	3.014	17.31	4	1
4	0.999	7.471	17.49	0	0
5	0.999	4.551	10.81	0	0
6	0.999	5.901	18.73	2	1
7	0.999	3.916	14.7	2	1
8	0.998	10.071	13.19	0	0
9	0.999	8.328	11.75	0	0
10	1.000	6.712	20.19	0	0

Abbreviations: CV: Coefficient of Variation. Dilution factor for all samples was 7.5.

Superoxide Dismutase

We used the Superoxide Dismutase Activity Assay from CellBiolabs, it is a colorimetric assay. It uses xanthine/xanthine oxidase system to generate superoxide anions. These anions generated by the system get detected by a chromogen solution, however in the presence of SOD the concentration of anions is smaller, reducing the color seen in the wells. The activity of SOD is determined by the inhibition of chromogen reduction.
